# Merging Datasets of CyberSecurity Incidents for Fun and Insight

**DOI:** 10.3389/fdata.2020.521132

**Published:** 2021-01-26

**Authors:** Giovanni Abbiati, Silvio Ranise, Antonio Schizzerotto, Alberto Siena

**Affiliations:** ^1^ Department of Social and Political Sciences, University of Milan, Milan, Italy; ^2^ Fondazione Bruno Kessler, Trento, Italy; ^3^ Department of Mathematics, University of Trento, Trento, Italy

**Keywords:** cyber security, data analysis, security incidents statistics, methodological framework, data breaches

## Abstract

Providing an adequate assessment of their cyber-security posture requires companies and organisations to collect information about threats from a wide range of sources. One of such sources is history, intended as the knowledge about past cyber-security incidents, their size, type of attacks, industry sector and so on. Ideally, having a large enough dataset of past security incidents, it would be possible to analyze it with automated tools and draw conclusions that may help in preventing future incidents. Unfortunately, it seems that there are only a few publicly available datasets of this kind that are of good quality. The paper reports our initial efforts in collecting all publicly available security incidents datasets, and building a single, large dataset that can be used to draw statistically significant observations. In order to argue about its statistical quality, we analyze the resulting combined dataset against the original ones. Additionally, we perform an analysis of the combined dataset and compare our results with the existing literature. Finally, we present our findings, discuss the limitations of the proposed approach, and point out interesting research directions.

## 1 Introduction

Cyber security incidents, such as intentional attacks or accidental disclosures, can have serious economic, social and institutional effects. The average total cost for companies and institutions spans from $7.35 millions in the United States to $1.52 million in Brazil, with a notable relation between the cost of the data breach and the number of lost records Ponemon Institute (2017). In this context, data about past cyber security incidents can give insights on potential vulnerabilities and attack types, thus helping to prevent them, provided that the data are available and have enough quality. Commercial reports on security incidents and data breaches can be easily retrieved; for example, statista (2018) is a well known online service that reports the annual number of data breaches and exposed records in the United States since 2005. While these reports are potentially interesting, the lack of transparency on the methodology used to generate them, as well as their (intended) non-academic audience, makes it difficult to use in scientific work. On the other hand, academic works that take a quantitative approach to the analysis of data breaches are less numerous. In Edwards et al. (2016), authors analyze data from the Privacy Rights Clearinghouse (PRC) and draw the conclusion that publicly reported data breaches in the USA have not increased significantly over the past 10 years, either in frequency or in size. Wheatley et al. (2016) combined two different datasets, DataLossDB (currently no more maintained as a public dataset) and the mentioned PRC, finding divergent trends between United States and non-United States firms. Xu et al. (2018) also uses the PRC dataset to analyze whether the data breaches caused by cyber attacks are increasing, decreasing, or stabilizing. Romanosky (2016) reports to have analyzed a commercial dataset of 300,000 observations about corporate loss events, having extracted a subset of around 15,000 observations about cybersecurity incidents out of it. As this last work confirms, having access to a commercial dataset seems to be a necessity since publicly available datasets are limited in size (up to 5,000 events, for the same years) and this reduces the effectiveness of several data analysis techniques.

To overcome this data availability limitation, we follow the intuition of Wheatley et al. (2016) to investigate on the possibility of combining several, publicly available, datasets and obtain a larger one, capable of supporting statistically grounded analysis of security incidents. A preliminary study on this idea has generated promising results, as reported in Abbiati et al. (2019). In a nutshell, the combination of multiple datasets has worked from a technical point of view, and a larger dataset has been generated. However, two main challenges remain to be properly addressed. First, the quality of the generated dataset, although apparently good, has not been validated. While some noise may have been introduced, it should not be statistically relevant, and the produced dataset should retain at least the same quality of the source datasets for the proposed methodology to be viable. The second challenge is related to some technical problems that have been noted ex post in the adopted methodology, which had an impact on the quality of the produced dataset. Such issues should have been highlighted by means of a validation step, but its lack led to unreliable results. Validating the proposed methodology has therefore become a primary requirement. The main hindrance to derive a merge dataset with good statistical properties is the well-known semantic heterogeneity problem (see, e.g., Halevy (2005)) deriving from the fact that independent parties building datasets for the same domain (such as data breaches) will end up using different (conceptual) schemas to arrange the information. Indeed, understanding the impact on the statistical significance on the merged dataset of the different strategies that can be used to manage the heterogeneity of the source datasets is a daunting task. This suggests a generate-check-refine loop in which, first a strategy for resolving semantic heterogeneity is proposed, statistical analysis is then applied, and if the results are not statically significant, another strategy is attempted. Indeed, for this approach to work, a mandatory pre-requisite is the availability of a tool-chain that provides an adequate level of support for the rapid generation of merged datasets that allow for the fine tuning of the strategies for heterogeneity management. The final dataset analyzed in this paper has been obtained through several refinements that contributed both to improve our understanding of the source datasets and gain experience in the difficult choices one is faced to resolve the problem of semantic heterogeneity. Concerning the statistical validation of the merged dataset, we use well-known techniques that allow to assess the quality of a merged dataset whose amplitude and scope is unprecedented when considering only publicly available sources; the only comparable dataset in size to ours is the privately owned one in Romanosky (2016). The reason for applying well-known statistical analysis techniques on the merged dataset is two-fold. On the one hand, our main goal is to validate the merged dataset; in this respect, results produced by traditional techniques are easier to interpret. On the other hand, although more sophisticated approaches are possible and attracting, we observe that those works (e.g., Edwards et al. (2016)) that have applied them contain forecasts that have failed to materialize. In other words, our emphasis is on being faithful to the source datasets with the aim of showing the added value of our methodology that enable the extraction of interesting insights in the merged dataset for the largest possible audience ranging from security experts to policy makers.

To overcome this data availability limitation, in this paper we follow the intuition of Wheatley et al. (2016), investigating on the possibility to combine multiple publicly available datasets to obtain a larger one, capable to support statistically grounded analysis of security incidents. A preliminary study on this idea has generated promising results, as reported in Abbiati et al. (2019). In a nutshell, the combination of multiple datasets has worked from a technical point of view, and a larger dataset has been generated. However, two main issues have emerged. On the one hand, the quality of the generated dataset, although apparently good, has not been validated. While some noise may have been introduced, it should not be statistically relevant, and the produced dataset should retain at least the same quality of the source datasets for the proposed methodology to be viable. On the other hand, some technical problems have been noted ex post in the adopted methodology, which had an impact on the quality of the produced dataset. Such issues should have been highlighted by means of a validation step, but its lack led to unreliable results. Validating the proposed methodology has therefore become a primary requirement.

To summarize, in this paper, we present a complete methodology for merging heterogeneous datasets of security incedents, addressing the issues emerged in Abbiati et al. (2019) and making some scientifically grounded claims out of the results. Specifically, the paper reports on two main activities. First, we present a revised version of the methodology, highlighting in particular the encountered issues, limitations and workarounds. Second, we analyze the generated dataset, with the twofold objective of extracting useful information and validate the output of the methodology, in particular with respect to the source datasets.

The paper is structured as follow. Section 2 describes our methodology to collect data and merge them into a single dataset. Section 3 presents the statistical analysis of the generated data and the produced results. Section 4 discusses the results with respect to the objectives and concludes the paper, outlining the future challenges.

## 2 Methodology

Quality of data is the base requirement for quality results, and the method used to produce data is an important factor on the way to improve data quality. Following our previous experience Abbiati et al. (2019), we organize the methodology in three phases: identification and collection (Section 2.1) of the datasets to be merged; mapping, merging, and selection of the original datasets into one possibly containing duplicates (Section 2.2); and redundancy elimination to filter out redundant entries in the merged dataset (Section 2.3). In a continuous learning-and-improvement cycle, we revise parts of the methodology to address all the identified weaknesses. In particular, we depart from our previous work Abbiati et al. (2019) in the following ways:

- the removal of the dataset from the ‘Information is Beautiful’ website^1^ as it does not contribute in any way to enrich the final dataset because of the tiny size and narrow focus (only the largest data breaches are considered);
- a complete rewriting of the redundancy elimination algorithm, which avoids some issues with the previous simplistic pairwise comparison method;
- a rethinking of the mapping adopted to harmonize the available datasets; and
- a different workflow, which allows us to take into account in the analysis not only the final dataset, but also the original ones.
Figure 2 shows an overview of the proposed methodology.

### 2.1 Identification and Collection

Cyber-security incident reports are published every day on the media. However, collecting systematically information about such incidents is problematic, maily because: 1) the information is distributed across a large number of websites, 2) it is exposed in natural language, and 3) older reports are often no more available. To mitigate these problems, there are initiatives that aggregate news about cyber-security incidents from third party sites as part of a professional work, making them available on-line as structured datasets.

Such initiatives rely on the activity of voluntary monitoring of information sources (such as online news or websites of authorities); or, on the spontaneous reporting of users about facts they read elsewhere. As a consequence, while some public datasets are large and contain up to several thousands of reports, they still may not provide a suitable coverage of the incidents reports. This possibility seems to be confirmed by comparing the size of the largest available public datasets (around 8,000 records, overall) to the declared size of the aforementioned commercial datasets (15,000 records). To have a wider coverage of the incidents’ reports, our intuition is that of further aggregating multiple datasets into a single and complete one, which may give us the opportunity to improve the (statistical) analysis of the trends in cyber security incidents.

This opportunity, however, does not come without challenges. Different datasets adopt different structures and are based on different classifications on key variables, such as the type of attacks or the economic sector of the firms affected. For this reason, the first phase of our methodolofy aims at developing a method to overcome the technical and conceptual heterogeneity in different source datasets.

**Identification**. We have identified in particular three main datasets of cyber-security incidents derived from three websites:

- **PRC**: Privacy Rights Clearinghouse—a United States-based nonprofit organization for privacy awareness and protection of individuals, which maintains a collection of data-breach records.
- **ITRC**: The Identity Theft Resource Center—a United States-based nonprofit organization, whose mission is to help victims of identity crimes (e.g., identity theft, scams, and frauds). In the context of a program to broaden public education and awareness in cyber-security and privacy issues, it provides a collection of data-breaches on yearly basis.
- **BLI**: The Data Breach Level Index—a website sponsored by Gemalto (which also offers cyber-security solutions), contains datasets of publicly disclosed data-breaches as well as related statistics with graphical representations. It also allows organisations to do their own risk assessment and suggests suitable mitigation measures aimed to reduce risks.

The reason to select the three datasets is twofold. On the one hand, while several annual reports on data breaches are elaborated by private companies and distributed on-line (e.g., Ponemon Institute (2017)), the original datasets and the methodology used to generate such reports are seldom made available with sufficient details.^2^ As a consequence, re-using the original datasets or the findings in the reports is extremely difficult in the academic context. On the other hand, PRC, ITRC, and BLI are freely available, the methodology used to build them is explicitly described, and—most importantly—some of them have already been analyzed with statistical methods in academic works Edwards et al. (2016); Wheatley et al. (2016); Xu et al. (2018). This provides sufficient guarantees on the quality of the datasets for their use in a scientific work and helps us understand whether enduring the pain of merging multiple datasets is worth the trouble and allow for better statistical results.

As reported in Table 1, the three datasets appear quite heterogeneous. They are made available in different formats (CSV, PDF, or HTML), the number of categories associated to incidents varies from 6 to 14, the number of incidents greatly differ—ranging from few hundreds to several thousands—as well as their time span. Additional sources of heterogeneity emerge as soon as we take a closer look. For example, considering the column ‘Attack type’ of Table 1, it emerges how PRC and BLI consider several types of attacks while ITRC focuses just on one type. Furthermore, the three used classifications differ in the number and types of classes of attacks. Worth to be noticed also that BLI contains a classification of the attackers, whereas ITRC and PRC are less granular.

**TABLE 1:** Descrition of the three datasets.

Id	Summary		Attack type	Organization type
BLI	breachlevelindex.com/data-breach-library		1. Identity theft	1. Education
	Format	Set of HTML pages	2. Account access	2. Entertainment
	Number of attributes	9	3. Financial access	3. Financial
	Number of entries	9,511	4. Existential data	4. Government
	Time range	2013–2018	5. Nuisance	5. Healthcare
				6. Hospitality
				7. Industrial8. Insurance
				9. Non-profit
				10. Retail
				11. Social media
				12. Technology
				13. Other
			
ITRC	www.idtheftcenter.org/data-breaches		1. Identity theft	1. Banking/Credit/Finance
	Format	Set of PDF files		2. Business
	Number of attributes	6		3. Educational
	Number of entries	9,743		4. Government/Military
	Time range	2005–2018		5. Medical/Healthcare
			
PRC	www.privacyrights.org/data-breaches		1. Payment card fraud	1. Bus.-Financial and insurance services
	Format	CSV	2. Hacking or malware	2. Bus.-Other
	Number of attributes	12	3. Insider	3. Bus.-retail/Merchant-including online retail
	Number of entries	8,611	4. Physical loss	4. Educational institutions
	Time range	2005–2018	5. Portable device	5. Government & military
			6. Stationary device	6. Healthcare, med. Providers & med. Insurance services
			7. Unintended disclosure	7. Nonprofits
			8. Unknown	8. Unknown

On the other hand, a lesser degree of heterogeneity is detectable on other domains, where the fields present a similar or identical schema or at least some conceptual similarity. For these reasons, harmonizing them into a single dataset looks challenging but feasible, and potentially useful.

**Collection**. The identified datasets do not expose APIs for querying them. A set of data collectors have therefore been developed to automatically retrieve the data from the Internet. Collecting the CSV files from PRC was straightforward, as well as collecting the HTML pages for BLI and parsing them to extract the data, required just a moderate additional effort for implementing the parser. The ITRC dataset, on the other hand, presented some additional challenges. First, the dataset is published as a set of PDF files, which are internally not well structured for being parsed. This requires to 1) extract the text from the PDF, and 2) identify appropriate pattern to detect, in the unstructured and mixed text fragments text string, the data entries and their fields. Additionally, the set of PDF files does not appear to have been generated automatically on the server at the time of the request, thus resulting in files released with slightly varying formats over the years, and at irregular intervals. For these reasons, the ITRC data collection has required two additional manual steps, namely 1) manually locate the PDF files in the ITRC website, and 2) compare the output of the automatic parsing against the PDF files, to ensure that parsing inaccuracies are detected and fixed.

### 2.2 Mapping, Merging, and Selection

**FIGURE 1:**
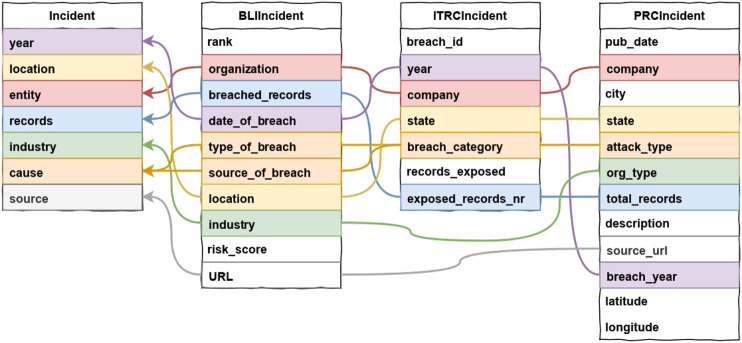
Redefinition of the data breach incident report.

In order to merge the three datasets into a single one containing all the incidents in each dataset, we define a fourth schema (unified schema), which will accommodate the data from the sources. The unified schema contains the information that are common to the three original ones, either directly—because the three datasets contain fields with the same semantics—or after the application of a suitable mapping—because, although the information is encoded differently, some fields represent the same information, that can be unified. The resulting schema contains seven categories: *Year*; *Location*; *Compromised records*; *Source*; *Entity*; *Industry*; and *Cause*. Figure 1 shows the mapping from the original categories to the harmonized ones. On the left-end side, our dataset schema is reported (“Incident”), while the colored lines define the mapping of each field to the source datasets. For example, the field “company” contained in a row of the PRC dataset (“PRCIncident” class, in the figure) has been found to be equivalent to the field with the same name in the ITRC dataset (“ITRCIncident”) and to the field “organization” in the BLI dataset (“BLIIncident”). These fields are represented in our schema as the field “entity.”

**FIGURE 2:**
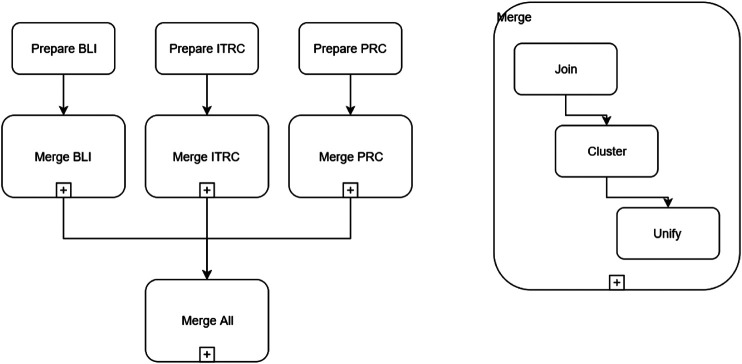
Overview on the dataset merge process.

The mapping is straightforward for the fields year of the incident, information source, target entity, location, and the number of compromised records because the values in such categories are homogenous across the three datasets. Much of the effort is needed to harmonize the type of organization (*Industry*) and the type of attack (*Cause*). More precisely, we need to perform the following two activities 1) mapping the original data and translating into two homogeneous classifications and 2) checking the homogeneity of the resulting dataset and merging into one or more sub-sets that show some internal coherence. We provide more details about these two activities below.

**Data mapping**. A critical work has been the reconciliation of the attack types, i.e. the *cause* field. While for some source categories the mapping was straightforward (e.g., Inside jobs), others made it difficult to produce a coherent and shared taxonomy of attacks. For example, BLI has two fields, “Type of breach” and “Source of breach”, which report information about what kind of data has been accessed (e.g., Financial data, Existential data) and the source of the breach (eg, Malicious insider, Hacktivist, State sponsored); PRC has a dedicated category for payment card frauds and differentiates various types of physical losses; ITRC puts in the same category physical losses and employee errors, while Improper Disposal is kept separated from an Accidental disclosure. We ended up with a custom classification, which attempts to minimize the number of categories. Specifically, the following categories have been identified: 1) two main categories for intentional disclosures: malicious attacks coming from inside (Insider job) and from outside (Hacking or Malware) activities; 2) one category for unintentional disclosures (Unintended disclosure); and 3) one residual category for other unmapped incidents (Other/Unknown). Attack types from the source datasets are assigned to one of these categories according to a case-by-case evaluation.

Another field that required reconciliation was the type of attacked organization, i.e. the field *Industry*. Source datasets classify organisations according to different taxonomies and with different level of granularity. A complete manual reclassification was therefore needed. We ended up defining a custom classification, which tries to optimize the coverage and distribute evenly the source categories. The adopted classification consists in the following macro business sectors: 1) Education and Healthcare; 2) Financial services; 3) Industrial production; 4) Information and Technology; 5) Other commercial activities; 6) Undefined privately-held businesses; 7) Public administration 8) Non-profit; 9) Other.

**Data merging**. We were then ready to merge the source datasets under the mapping defined at the previous step. This is a complex operation that has the purpose of reducing one or more input datasets into one output dataset, preserving the quality of the data. Specifically, merging is composed of three main tasks:

- **Join**: multiple datasets having the same format are piled together into a single dataset;
- **Unify**: clusters are parsed and it is decided whether the rows of each cluster represent the same event or they are just false positives. If they are recognized to be multiple instances of a same event, one of the rows of the cluster is picked as arepresentative and the others deleted. Otherwise, the cluster is released.

In order to produce the final dataset, merging has been applied repeatedly: to the source datasets, separately, to join all years, cluster and unify them (to detect, for example, data breaches reported more than once by different users); afterward, the results of merging each dataset have then been merged into a single dataset.

**Data selection**. At the end of the data merge step, we derived a single dataset containing 18,030 rows. After an analysis of the entries, it resulted that the largest number of incidents concerned organisations or companies located in North America (either United States or Canada). Specifically, slightly more than 90% of the incidents occurred to organisations or companies in North America. This is probably due to two factors: 1) the dataset were taken from online services located in United States and 2) these countries (United States and Canada) are subject to laws and regulations with mandatory requirements for the notification of security breaches since 2004/2005 or earlier. We hence decided to limit our analyses only to incidents happened in North America.

### 2.3 Redundacy Elimination

Despite showing some internal coherence, the dataset obtained after mapping and selection shows some redundancy because of the same event reported twice or more; this is the result of merging three datasets. In fact, while the datasets focus on slightly different kind of data breaches, they present some overlaps in the reported events. Apart from a technical issue, this fact represents also a major threat to the validity of our approach, because if the overlapping is too large, the datasets can simply be considered duplicates of each other and there is no hope that the merged dataset allows for more precise statistical analyses of the data breaches. For this reason, we have defined a cleanup procedure, which we describe below together with the techniques that we use to detect and eliminate duplicates.

**Duplicated events**. Syntactic redundancy refers to two or more incidents represented as records with identical fields. This can be easily detected and removed, but an issue emerges on the actual definition of “duplicate.” In many cases, two or more records differ only for minor elements; to human judgment they are clearly the same, they should be detected as duplicates and reduced to a single record. Hence, the identification of “likely duplicates” becomes the key challenge. For example, in some cases two rows reported a security incident in the same year, concerning the same entity, but only in one case the number of compromised records was known. A check on a sample of original incident URL sources suggested that these instances referred actually to the same events. Such cases were removed, maintaining the records reporting the number of compromised records only. The trickiest case referred to records with similar entity names. Different sources reported the same incident recording the entity name with different acronyms, shortcuts, legal specifications and mistakes. We hence identified an additional set of “likely duplicates” that we briefly describe below:

*Differently decorated names*. Differences could be related to partial omissions, typically concerning legal specification (e.g., “Google” and “Google, inc.“). In this case the two names were unified through a simple catalog of pattern templates.

*Similar names*. A more problematic set of cases was due to entity names that were actually similar but no precise detection rule could be defined. These cases involved typically spelling or punctuation mistakes (e.g., “HOMECARE OF MID-MISSOURI INC.” and “HOME CARE OF MID MISSOURI”, or “COHN HANDLER STURM” and “COHN HANDLES STURM”). These are basically singletons and defining pattern matching for all the instances would have resulted in a huge but useless effort. To overcome this problem, an algorithm has been applied to spot similar entities.

The algorithm worked by comparing all the possible (n(n−1))/2 pairs of entities, assigning to each pair a score. A graph was built, in which each vertex corresponds to a dataset row and each edge between vertices measures the similarity distance between the rows along multiple aspects: entity similarity, compromised records cardinality, year, and so on. Subsequently, the graph was queried, identifying clusters of similar rows. For example, two rows, in which the difference in the number of exposed records is less than 5% (e.g., 1,000,000 and 1,000,615) are considered having the same number of exposed records. For the entity name, the similarity is calculated using the Jaro-Winkler distance.^3^ The algorithm was fed with a threshold value of 0.18. This threshold was identified through a manual process. It was carried out under the hypothesis that 1) if the threshold is too low, the number of false negatives grows; to the extreme point, a zero threshold implies that no duplicates are identified, and the number of false negatives is maximum. 2) if the threshold is too high, the number of false positives grows; to the extreme point, a threshold value of 1 means that all the entries are considered duplicates, and the number of false positives is maximum. 3) between 0 and 1, the number of false negatives decreases, while the number of false positives increases, and there is a point in which the falses (positives plus negatives) reaches a minimum. If hypothesis 3) holds, we can identify the trade-off point, which is our threshold. Otherwise, this approach should be rejected. Some manual tests showed that hypothesis 3) holds. Having selected a sample of 50 pairs, the duplicate identification algorithm was executed, with different, coarse-grained (i.e., with intervals of 0.5) threshold values. The range 0.1–0.25 was quickly identified as the minimum region, thus confirming in first place hypothesis (c). A more fine-grained search was more difficult, because in this region, with small changes in the threshold value, it turned out that many local minima/maxima existed. Additionally, the quality of the sample was clearly contributing to the evaluation of the quality of the algorithm. The threshold was therefore selected by taking into account the cases, within the sample, which were considered more critical. Rows with a distance lower than this threshold were considered concerning the same entity. Lastly, for the *Year* field, two rows are considered similar only if the year is the same. Rows, whose distance scores under a target threshold for all the aspects, are considered duplicate rows.

*False positives*. They were a major issue in the activity of identifying similarities. Particularly problematic was the case of entities sharing part of the name but indicating different institutions (e.g., “University of … “). This phenomenon had to be contrasted with cases of true positives, such as different branches of a same organization, such as territorial units (e.g., “7eleven’ York”, “7eleven Baltimora”).

*False negatives*. Similarly to the case above, also false negatives require human intervention and cannot be easily translated into clear-cut rules. Examples are “Google” and “Alphabet”, in which the latter is the new corporate name of the first; or “UNIVERSITY OF CALIFORNIA LOS ANGELES” and “UCLA”, where the latter is an acronym for the first.

Once the algorithm has generated the list of candidate duplicates, a manual pass allowed us to identify false positives. To deal with them, a further catalogs of patterns (white list) have been defined listing candidate duplicates. More difficult was to deal with false negatives. So far, they are added to a third pattern catalog (black list) whenever they are identified.

*Legal entities*. A last issue concerns the legal setting of reported entities. Companies and public administrations can be articulated in hierarchies of controlled companies. Controlled companies can have legal personality, and therefore their own name, which in some cases may differ completely from the original. If the same security breach is reported multiple times and using different names (of the controller and controlled company), this is not easily identifiable in an automatic way.

### 2.4 Merged Dataset

The merged dataset, as resulting from the described process, contains 16,340 entries. We can not claim the resulting dataset to be fully duplicate-free, also because duplicates exist in the source datasets (13% in PRC, 8% in BLI and 9% in ITRC). The application of the algorithm to the dataset resulting from unifying the source datasets as explained above allowed us to spot about 28% of duplicate events. Interestingly, we managed to fill in missing values of some incidents by combining information from different datasets (i.e. for the same event, we used the number of records in PRC and the cause of the attack in BLI) in about 2,800 instances when the missing information was the cause of the attack and in about 1,300 cases when the number of compromised records was unknown. These results indicate that the sources are only partially overlapping and that data inspection and management can increase the quality of existing publicly available data sources, for example by reducing the problem of incomplete entries.

We also inherit other aspects of the source datasets: firstly, the dataset contains only attacks reported to authorities, which do not include foiled or unreported attacks; secondly, there are many important information (such as technologies used by firms and public administration departments) about which nothing is known. Finally, our mapping of the categories of attacks and organisations remains somehow arbitrary, but unfortunately arbitrariness also affects the source dataset, as none of the publisher adopted an official classification, using rather their own.

## 3 Data Analysis

**FIGURE 3:**
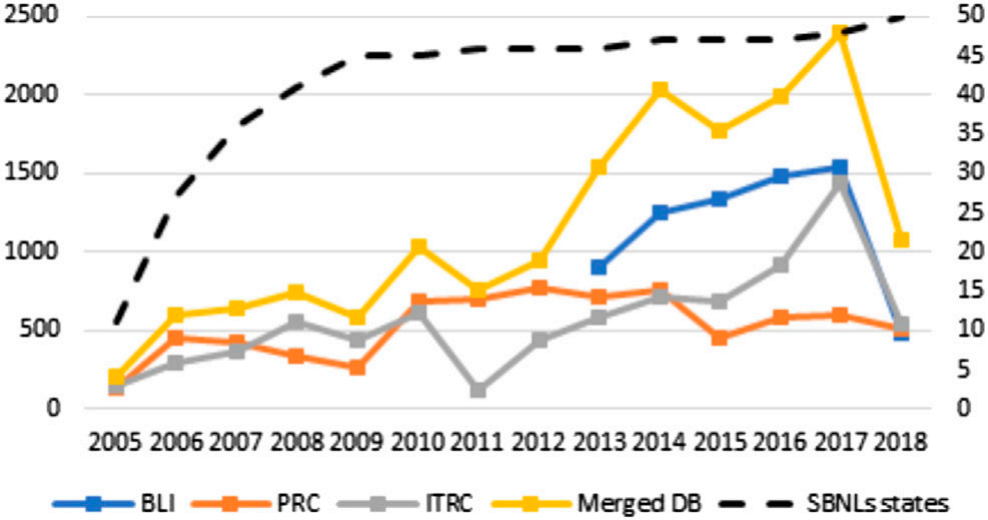
Yearly reported number of cyber attacks by database and number of United States adopting SBNLs–2005/2018.

**FIGURE 4:**
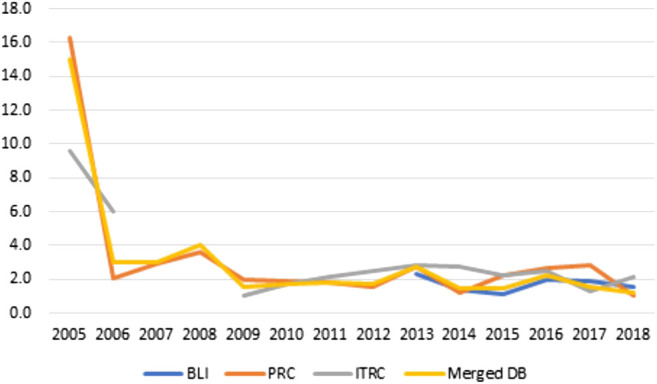
Yearly median number of compromised records by database–2005/2018.

**FIGURE 5:**
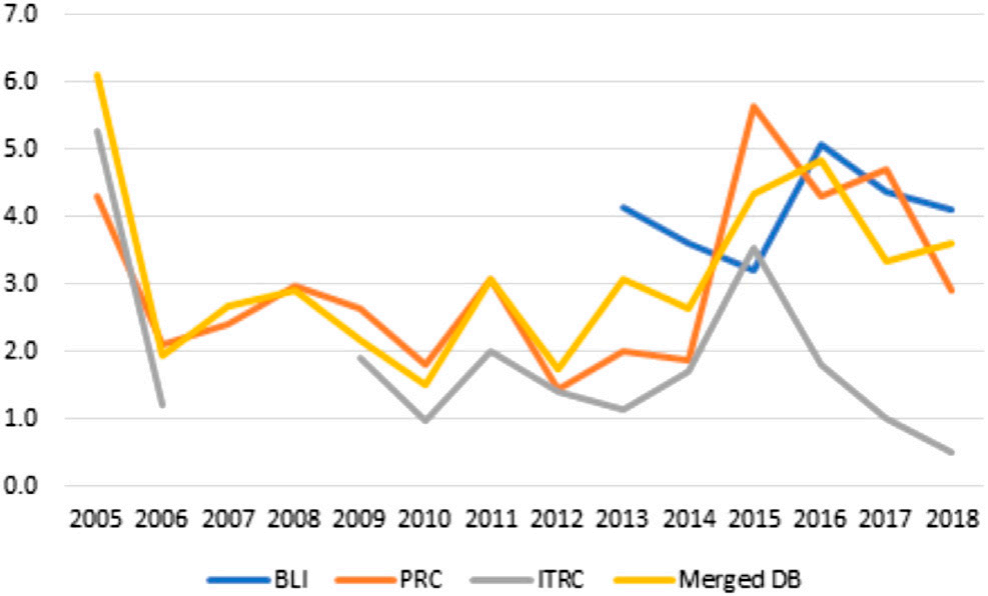
Yearly percentage of highly disruptive attacks by database–2005/2018.

The resulting dataset has been analyzed against the original ones (BLI, ITRC and BLI), to obtain some statistical measures about the cyber-security incidents trends, as well as to validate the resulting data. We start the analysis by plotting the trends of the number of cyber attacks and a measure of the damage they caused over the period 2005–2018 (Figures 3–5).^4^ Figure 3 starts by reporting the number of unique events (post-cleaning) contained in each database (left *Y* axis) and the US states adopting Security Breach Notification Laws (SBNLs hereafter, whose number is depicted on the right *Y* axis). It is possible to highlight four main results from Figure 3. First, the reported number of attacks constantly increased from 2005 onwards,^5^ leading to an increase of 1,000% in the time-span considered. This increase can only partially be attributed to the merge of different sources: incidents recorded in PRC increased by 350%, and those recorded in ITRC by about 850%. Second – and important –, the increase in the number of attacks seems to be independent, at least in recent years, from the spread and the enforcement of SBNLs. In other words, the growth we are observing does not depend on the obligation for firms to provide data, since such laws were already in place in 45 States in 2009 (for the impact of notification laws in earlier years see Nieuwesteeg (2013)). Third, the databases seem to be only partially overlapping: the number of records in the merged database is on average 20 to 34% lower of the algebraic sum of the three sources. It is also worth noting that the three databases are not registering the exact same events, and this confirms the necessity for a detailed procedure for duplicates identification when dealing with the merge of separate sources. Fourth, we can appreciate three main discontinuities in the trends. The first occurred in 2010, when the number of attacks doubled in comparison to the previous year. The second is visible with the introduction of the BLI database in 2013. The last one seem to be occurring in 2017, with a new spike in incidents that seem to be mainly driven by the records contained in ITRC.

Figures 4, 5 report for the same time-span the same measure of damage (number of compromised records), but focusing on different aspects of its distribution: the median and the percentage of highly disruptive attacks, defined as attack involving over 1 million records. We choose the focus on such distributional quantities because the database is characterized by a low but non-negligible number of extreme events (attacks involving millions or billions of records) that heavily affect the mean but not the median or their quota. In case of Figure 4 we can see no clear-cut trend. 2005 opens the series with a considerable value in the median number of attacks, a number that decreases rapidly in the subsequent years along with a parallel increase in the number of attacks. After 2008 the median number of compromised records fluctuates around 2,000, a value that can represent a considerable damage for the medium-to-small businesses and public organizations^6^.

Figure 5 shows the percentage of highly disruptive attacks by year and source. The high share of such attacks in 2005 may have been, we hypothesize, the triggering event that lead to the creation of public databases investigating and monitoring the phenomenon. After the spike in 2005 we observe a stable percentage of disruptive attacks until 2013 (1–3% according to the different source), and a strong, though somehow unstable, increase from 2014 onwards, that posits the average share above the threshold of 3%. It is however risky for the moment to affirm that we are in front of a change in the strength of the attacks by virtue of the high differentiation of this piece of data by source, the non-negligible number of missing values and the volatility of the phenomenon. In light of this evidence we would be very cautious in sharing the “optimistic” methodology produced by Edwards et al. (2016), according to which future tendencies about cyber-attacks could be inferred by previous trends. Contrarily to the authors’ predictions of a decrease of the damage posed by cyber-attackers in 2015/2017, the last 3 years have been plagued by some of the most devastating attacks ever recorded.

The following tables break down the information on the number of attacks by year and sector (Table 2) and their relative significance (Table 3). Given that the number of missing values on attack type and sectors have been reduced during the process of databases merging (see methodological section), we will present elaborations on the merged database only. According to Table 3), the sector mostly damaged by cyber-attackers is health and education (43.1%). Commercial activities account for a fifth of the observations (21.4%). Sectors such as public administration, and financial services are also a typical target for cyber attackers (9–13%). At the bottom of the ranking stand information and technology (2.9%), industrial production (0.5%) and no-profit (0.8%). For a residual 4% of attacks the activity sector was not coded in the original source and it could not be recoded afterward. The ranking depicted above is constantly evolving: health and educational organizations’ quota is decreasing from the period 2011–2014 (values often above 50%) to the 36.3% registered in 2017. The quota of attacks suffered by public administration has decreased rapidly in the period 2006–2011 and then remained stable. For other sectors it is more difficult to trace a trend. Information and technology, the sector likely to be more subject to attacks, holds a quota relatively small.

**TABLE 2:** Attack by year and sector (Percentages).

Year	Education and healthcare	Financial Services	Industrial Production	Information and technology	Other Commercial Activities	Undefined Private Sector	Public Secto	No profit	Unknown	Total
2005	52.7	15.6	0.0	0.0	15.6	1.5	13.7	1.0	0.0	100
2006	34.0	17.6	0.0	0.0	16.2	5.0	26.4	0.8	0.0	100
2007	33.7	13.8	0.0	0.0	23.2	5.1	22.4	1.9	0.0	100
2008	34.8	10.4	0.0	0.0	31.2	3.6	18.8	1.2	0.0	100
2009	35.0	10.1	0.0	0.0	32.3	2.6	19.0	1.0	0.0	100
2010	44.6	9.7	0.0	0.0	28.1	2.1	15.0	0.6	0.0	100
2011	53.1	6.4	0.0	0.0	16.2	11.3	11.1	1.8	0.0	100
2012	49.4	7.0	0.0	0.0	24.6	6.2	11.2	1.6	0.1	100
2013	48.7	8.3	0.0	3.8	17.8	1.8	12.1	0.3	7.3	100
2014	55.1	7.3	0.0	4.1	14.6	1.6	12.0	0.1	5.3	100
2015	40.6	10.7	0.0	4.1	21.6	2.8	12.1	0.1	8.0	100
2016	43.2	8.8	1.0	6.3	22.3	2.5	10.9	0.9	4.1	100
2017	36.3	9.7	1.3	3.3	35.7	4.7	7.3	0.5	1.3	100
2018	34.3	12.5	1.5	2.2	5.5	20.7	5.7	0.5	17.0	100
Total	43.1	10.2	0.5	2.9	21.4	4.6	12.5	0.8	4.0	100

**TABLE 3:** Relative risk for a firm of being attacked, by sector.

Year	Education and healthcare	Financial Services	Industrial Production	Information and technology	Other Commercial Activitie	Undefined Private sector	Public Sector	No profit	Unknown	Total
2005	52.7	15.6	0.0	0.0	15.6	1.5	13.7	1.0	0.0	100
2006	34.0	17.6	0.0	0.0	16.2	5.0	26.4	0.8	0.0	100
2007	33.7	13.8	0.0	0.0	23.2	5.1	22.4	1.9	0.0	100
2008	34.8	10.4	0.0	0.0	31.2	3.6	18.8	1.2	0.0	100
2009	35.0	10.1	0.0	0.0	32.3	2.6	19.0	1.0	0.0	100
2010	44.6	9.7	0.0	0.0	28.1	2.1	15.0	0.6	0.0	100
2011	53.1	6.4	0.0	0.0	16.2	11.3	11.1	1.8	0.0	100
2012	49.4	7.0	0.0	0.0	24.6	6.2	11.2	1.6	0.1	100
2013	48.7	8.3	0.0	3.8	17.8	1.8	12.1	0.3	7.3	100
2014	55.1	7.3	0.0	4.1	14.6	1.6	12.0	0.1	5.3	100
2015	40.6	10.7	0.0	4.1	21.6	2.8	12.1	0.1	8.0	100
2016	43.2	8.8	1.0	6.3	22.3	2.5	10.9	0.9	4.1	100
2017	36.3	9.7	1.3	3.3	35.7	4.7	7.3	0.5	1.3	100
2018	34.3	12.5	1.5	2.2	5.5	20.7	5.7	0.5	17.0	100
Total	43.1	10.2	0.5	2.9	21.4	4.6	12.5	0.8	4.0	100

These figures alone, though, cannot provide a meaningful picture of the exposure of activities to cyber-attacks. For this reason we calculated the odds of being target of a cyber attack by sector and year. Odds ratios describe the relative risk of being attacked in relations to the sector in which the firm operates. The situation is depicted in Table 4. Industry and other commercial activities have a very low incidence rate all throughout the period, probably given the small relevance of personal data storage in their activity. Health and education firms show very high values, but a declining trend in the odds of being attacked since 2014. As predictable, financial services are a relatively common target for cyber-attackers (odds ratios around 2/5 for all the period considered). Information and technology firms represent a very interesting case: they have both a low percentage of attacks and have an incidence rate high, but definitely lower than educational and financial firms. This result is due, we hypothesize, in part because of inaccurate coding and in part because of the higher value that Information & Technology firms put in prevention measures. Nonetheless, cyber-attacks on IT and technology firms are often of devastating scale, when they occur: in the 26% of cases, attacks on IT and tech firms involved more than 1 million records, compared to much lower figures in other sectors (0.1%/5%) and the median number of stolen records amounts to 39,000, as compared to an average comprised in a range of 1,000/2,000 stolen records in other sectors.

**TABLE 4:** Type of attack by year (percentage).

Year	Hacking or Malware	Inside Job	Unintended Disclosure	Other/ Unknown	Total
2005	26.3	6.3	34.2	33.2	100
2006	13.4	5.0	54.2	27.4	100
2007	11.0	3.6	43.7	41.7	100
2008	7.9	4.1	26.3	61.7	100
2009	10.1	5.7	22.0	62.3	100
2010	10.7	9.9	26.3	53.1	100
2011	22.1	11.0	30.0	36.9	100
2012	27.1	8.6	25.4	39.0	100
2013	38.9	13.4	27.3	20.4	100
2014	45.7	13.4	23.8	17.1	100
2015	51.8	10.5	23.6	14.2	100
2016	59.9	6.2	21.0	12.9	100
2017	54.1	5.4	15.8	24.8	100
2018	28.9	2.1	14.1	54.9	100
Total	37.4	8.2	24.6	29.8	100

Table 4 illustrates the attacks by type. The trends depicted show the rapidly changing geography of the way cyber-attacks are conducted: an increasing majority of attacks are conducted via hacking and malware, along with the diffusion of online tools to work and store data. Their share of total attacks shows a spectacular increase, especially in the period 2008/2015, when they passed from being a residual 7% of attacks to the absolute majority (51.8% in 2015 and almost 60% in 2016). At the same time, unintended disclosures are losing importance, being the modal cathegory before 2008 and now representing about the 16% of incidents. The increasing awareness about the vulnerability of IT systems and the large scale implementation of security measures may explain this decreasing trend. Finally, inside jobs are always present in percentages fluctuating from 5% to 14% across sectors and across years, without showing a clear trend. These results do not necessarily invalidates the findings in Verizon, 2018a and Verizon, 2018b that estimate inside jobs at around the 23% of total attacks with a peak of 56% among healthcare organizations: first, a significant minority of attacks is of unknown origin and it may well fall into this category; secondly, we cannot parcel healthcare and education out in our data. The “other/unknown” category is currently the modal one and we conjecture that this data has two different explanations: the first refers to the ability of cyber attackers. The smoother is the attack, the more difficult it is to identify, and then report, its actual cause (and, relatedly, the size of the damage caused). The second one refers to the quality of the data. A part of it may be in fact attributable to sloppiness in reporting the attacks. We have indirect evidence of it when cross-tabulating the sector with the type of attack (Table 5): the “unknown” category (perhaps another example of sloppiness) is the one for which about 46% of the attacks are of unknown origin. As concern the rest of the sectors, it is interesting to notice how hacking and malware represents by far the main problem in all technology intensive private sectors (Information & Technology, Financial services, Industrial Production), while for the others the risk represented by hacker is paired by the one coming from unintended disclosures, maybe as a consequence of their lower investments in IT training and related procedures.

**TABLE 5:** Type of attack by sector (percentage).

	Hacking Or malware	Inside Job	Unintended Disclosure	Other/Unknown	Total
Education and healthcare	33.6	8.2	31.5	26.8	100
Financial services	44.4	10.4	23.2	22.1	100
Industrial production	82.1	9.4	8.5	0.0	100
Information and technology	75.3	4.9	19.5	0.5	100
Other commercial activities	49.2	7.5	34.2	9.1	100
Undefined private sector	42.8	7.4	9.9	39.9	100
Public sector	58.9	7.6	23.7	9.8	100
No profit	29.0	10.6	32.1	28.1	100
Unknown	39.1	4.9	10.6	45.6	100
Total	37.5	8.2	24.6	29.7	100

## 4 Discussion and Conclusion

This paper presents our findings in the analysis of historical trends in cyber-security incidents. Our work rely on a procedure of datasets identification, collection, mapping, merging, selection, and redundacy elimination, which allowed us to build a large dataset of cyber-security incidents by unifying a collection of three publicly available datasets of different size and structure. This way, we overcame the lack of publicly available datasets of substantial size observed in previous research Romanosky (2016).

By analyzing the resulting dataset with standard statistical techniques, our work confirms the generally observed rapidity with which the phenomenon of cyber-attacks is evolving. While incidents caused by malicious outsiders passed from 16% to 50% in a time-span of just 5 years, other leading causes of data breaches such as malicious insiders and unintended disclosures lost most of their importance in the same period. There may be multiple causes underlying this trend. On the one hand, the decreasing relevance of unintended disclosures and malicious insiders may be the result of the adoption of better security procedures and awareness programs by companies and organisations. On the other hand, remote attacks are more and more widespread because of the explosion of personal and sensitive data available on-line resulting from the digitalization of many aspects of our lives. These factors seem to confirm the idea that organisations and companies should take a holistic approach and tune their cyber-security postures according to a variety of sources about threats and countermeasures including cyber-intelligence information about current threats provided by, e.g., national or international Computer Emergency Response Teams (CERTs). It is thus not surprising that the forecasts about the size of 2015 and 2016 data breaches contained in Edwards et al. (2016) remain partly unachieved.

Concerning the limitations of our approach, two issues must be considered. The first is related to the coverage of data and is shared with previous work (e.g., Romanosky (2016)). Since the three datasets used to build ours are based on public notifications to authorities, it is unclear whether the data are representative of the overall phenomenon of cyber-attacks or not. We draw this consideration from the comparison of two figures. In our dataset, the share of private United States companies and organisations involved in security breaches amounts to minuscule figures, namely 0.02% (or less) per year. An official report based on a representative United Kingdom sample highlights that 67% of medium-large firms have suffered from cyber-attacks in 2016 Klahr et al. (2017). The corresponding number for Italy in the same period, based on another national representative survey Biancotti (2017), is 43%. Future research should focus on gathering additional sources of information to understand to what extent our analyses reflects actual trends operating in the overall population of United States firms and organization. The second issue to be considered is the remarkable amount of effort required to make the merged dataset coherent and uniform. The result is apparently worth the effort; a database derived from publicly available information that is comparable in size to that used in Romanosky (2016), which is privately owned and contains around 15,000 descriptions of data breaches. However, we acknowledge that the relevance of the results depends on the quality of the generated dataset, which in turn depends on the quality of the method used to join the source datasets: it must be able to eliminate redundancies and consistently map the source categorisations into one which is general enough to accommodate those used in the initial datasets and—at the same time—not too coarse to loose precision and significance in the analysis phase. To tackle this issue, our current efforts are devoted to reach a high-level of automation of the various steps of the methodology by developing a toolkit for automatically collecting, tidying, mapping, and merging datasets of cyber-security incidents. The main benefit of developing such a toolkit is flexibility along two dimensions. First, it will be possible to experiment with different taxonomies for the types of attacks and economic sectors to better identify which option minimizes the loss of precision and coherence when merging different datasets. Ultimately, this would reduce the level of arbitrariness in the data manipulations besides those imposed by the publishers of the original datasets. The second dimension is a tighter integration with the data analysis phase: depending on the results of the latter, we can decide to investigate some features of the component datasets and use the results to fine-tune some aspects of the collection, selection, mapping, and redundancy elimination steps. The flexibility deriving from a high-level of automation of the methodology will also simplify the inclusion of new datasets, increase the size of the merged dataset, and possibly make the application of a wider range of data analysis techniques.

The difficulties we faced in merging the datasets are well-known in the database, XML, web service, and ontology communities in which a long series of works have been devoted to solve the problem of semantic heterogeneity for building applications that use multiple sources of information; see, e.g., Halevy (2005) for an introduction to the problem and an overview of some solutions. The problem stems from the observation that when database schemas for the same domain are developed by independent parties, they will almost always be different from each other and reconciling such heterogeneity requires both domain and technical expertize; the former to understand the meaning of the datasets together with the purpose of their merging and the latter to design and implement the necessary transformations of the source datasets. It is not by chance that the second and last authors of this paper are researchers in cybersecurity whereas the first and the third authors are experts in statistics. The main difference between our work and those available in the literature is with respect to the purpose of merging datasets; our goal is to support more precise statistical analyses whereas that of the related works is to answer queries across multiple sources of information. Despite intensive research, few solutions are available to the problem of semantic heterogeneity and most are inherently heuristics, human assisted processes; see, e.g., Rahm and Bernstein (2001) for an overview of classical approaches to the problem. The crux of these works is to define heuristics that assist humans in defining a mapping among the various elements of the schemas associated to the source datasets while providing a high degree of automation to implement the transformations based on such mappings. Our methodology shares the same spirit by requiring the definition of a data mapping (recall the paragraph with the same name in Section 2.2) and then providing a high degree of automation for the remaining phases. An interesting and recent approach to partially automate the definition of schema mappings uses Machine Learning algorithms (see, e.g., Cappuzzo et al. (2020)); it seems a promising line of future work also in our context.

It is well-known that semantic heterogeneity is exacerbated by two factors Rahm and Bernstein (2001): 1) the presence of semi-structured data because of the high flexibility of partially defined schemas and 2) the possibility that different data values denote the same object (this is called data heterogeneity). We observe that both factors are present in our work; for (a), recall the discussion about the ITRC dataset in the paragraph **Collection** at the end of Section 2.1 and for 2) see the paragraph **Duplicated events** in Section 2.3. For issue (a), namely dealing with semi-structured data, it is crucial to extract some structure to support the definition of schema mappings from the source datasets to the merged one. The problem of discovering structure in semi-structured data is known in the literature—see, e.g., Nestorov et al. (1998)—to support presenting and querying the dataset. Unfortunately, the approach in Nestorov et al. (1998) does not seem to be applicable to our context as it assumes labeled, directed graphs as the underlying semistructure of the datasets. This is not our case as we work with tables whose columns contain cells with heterogeneous content. More similar to our approach is the work in Cavalcanti et al. (2017) whose goal is to merge semistructured datasets by reducing the integration effort while guaranteeing the quality of the resulting dataset. The main finding of Cavalcanti et al. (2017) is the observation that conflicts (eg, those derived by duplicates that went undetected) in the merged dataset should be minimized at all costs, even by sacrificing the granularity of the available information. This agrees with our experience; for instance, we decided to forget certain fields in some of the original datasets (see, e.g., records_exposed of the ITRCIncident dataset or risk_score of the PRCINcident dataset in Figure 1) in the definition of the schema mappings for the sake of maintaing a symmetric contribution from each one of the source dataset and thus avoid possible conflicts in the merged dataset.

For tackling issue (b), namely data heterogeneity, it is crucial to identify appropriate data cleaning techniques; see, e.g., Rahm and Hai Do (2000) and Ridzuan et al. (2019) for overviews of traditional techniques and extensions for handling big data, respectively. In general, the elimination of noise (of which redundancy is one of the most prominent aspects) is recognized as fundamental for obtaining high-quality datasets and, at the same time, is an interactive, error prone and time consuming activity. As a result, assessing the quality of the data generated by the data cleaning techniques and its fitness for the purpose (in our case, statistical analysis) becomes very difficult to evaluate (if possible at all). For this reason, we have taken a very pragmatical approach by adopting a generate-check-refine loop in which, a merged dataset is generated according to a certain strategy for addressing the various aspects of the semantic heterogeneity problem, the result is statistically analyzed, and if the outcomes of the analysis are not statically significant, the strategy is refined and another iteration of the loop is performed. More sophisticated approaches can be attempted to cope with duplicates, such as those in Hassanzadeh and Miller (2009) whose idea is to keep duplicates in probabilistic databases that can return answers to queries with the associated probabilities of being correct. As explained in Hassanzadeh and Miller (2009), for this to work in practice, sophisticated clustering algorithms needs to be used in place of standard thresholding techniques (such as the one used in this work based on the Jaro-Winkler distance) for eliminating duplicates. It would be interesting to integrate this and similar techniques such as Mishra and Mohanty (2020) that use unsupervized learning algorithms to increase the level of automation of the redundancy elimination step, which—as already observed above—is one of the most demanding in terms of time and user intervention of our approach.

We also mention that other attempts at defining suitable procedures for dataset merging have been proposed in different application contexts ranging from data warehouses to GIS applications; see, e.g., Olaru (2012) and Suryana et al. (2009). While interesting, these works propose merging strategies that are domain specific and seem to be difficult to be reused in our context.

The work described in this paper raises an important observation. As stated in Section 1, several surveys and statistical reports are available online, mostly from private companies. Since the issues we reported depend only partially from our approach, it should be argued that the reports available online suffer the same limitations and issues. This calls for a deeper scientific exploration of the available data, to better evaluate the quality of the dataset and make transparent and questionable the results, including cross-fertilization from other research communities as those discussed above addressing the problem of semantic heterogeneity.

To cope with this issue, our efforts are devoted to allow a range as wide as possible of professionals involved in managing cyber-security (such as technologists, insurers, or policy makers) to have an insight on the data and methods presented here, and to work on them. As a first concrete step to promote the use of our datasets and methodology, we provide pointers to the on-line datasets and the merged one, plus additional material. We will also provide adequate tool support to the methodology described in this work and perform more extensive investigations about the datasets considered here and others that will be made available to us.

## Data Availability Statement

The datasets generated for this study are available on request to the corresponding author.

## Author Contributions

All authors have made a substantial, direct, and intellectual contribution to the work and approved it for publication.

## Conflict of Interest

The authors declare that the research was conducted in the absence of any commercial or financial relationships that could be construed as a potential conflict of interest.

## Footnotes

1
https://www.informationisbeautiful.net/visualizations/worlds-biggest-data-breaches-hacks/.

2 One notable exception is the production of annual Data Breach Investigation Reports by Verizon available at https://enterprise.verizon.com/resources/reports/dbir. A dataset of security incidents is available at https://github.com/vz-risk/vcdb but it is only an excerpt of that used to generate the annual reports because Verizon ability to share more comprehensive information is limited by agreements with partners and customers to which the data breaches and security incidents relate.

3 The Jaro-Winkler algorithm Porter et al. (1997) is one of the most commonly used algorithms for evaluating string similarity, and depends on the weighted percentage of transpositions required to go from one string to another.

4 At the time of writing, the publicly available data from the three datasets extends from January 2005 to June 2018. More precisely, data from ITRC and PRC cover up to 2019 whereas BLI only up to June 2018. For this reason, we limit our analysis to June 2018.

5 Note that the partial drop in 2018 is due to the fact that the data refer to the first 6 months of the year.

6 Results about compromised records have to be taken with caution. Despite the analysis is being performed on the results of merging three datasets, values on the damage caused by cyber-attacks are missing for 35% of the entries.
